# English vowel identification in noise shows reduced native-language influence: a behavioral study using machine-learning analysis of Chinese children

**DOI:** 10.3389/fpsyg.2026.1952327

**Published:** 2026-09-24

**Authors:** Qiuhui Wang, Lin Mi

**Affiliations:** 1 School of Information and Communication Engineering, North University of China, Taiyuan, China; 2 The Affiliated Brain Hospital, Guangzhou Medical University, Guangzhou, China

**Keywords:** acoustic background, Chinese children, English vowel identification, machine learning, vowel identification

## Abstract

**Introduction:**

Non-native listeners experience greater difficulty perceiving second-language (L2) speech in noise, yet whether first-language (L1) phonological skills continue to support L2 perception under adverse acoustic conditions remains unclear.

**Methods:**

We examined English back-vowel identification in 52 Chinese fifth- (*n* = 25) and sixth-grade (*n* = 27) children under quiet, 5 dB SNR, and −5 dB SNR conditions. Chinese vowel identification in quiet was used to index L1 ability, and linear regression and random forest models were used to characterize associations among acoustic background, grade, and L1 perception.

**Results:**

Sixth graders outperformed fifth graders across conditions, but the grade advantage decreased as noise increased, with accuracy differences of 13.30%, 9.80%, and 3.20%, respectively. Chinese vowel identification significantly predicted English vowel identification in quiet but showed no significant predictive association under either noise condition. Random forest analyses complemented the specified linear model by revealing nonlinear predictive patterns, including a grade-by-noise interaction not detected by the linear model in this sample. These exploratory findings should be interpreted cautiously given the modest sample size (*N*= 52) and strict participant-level validation.

**Discussion:**

The findings suggest that the association between quiet-measured L1 vowel identification and L2 vowel identification is context-dependent and attenuates under noisy listening conditions. They also indicate that grade-related advantages diminish as acoustic difficulty increases, while machine-learning analyses may provide complementary information about predictive patterns without establishing underlying cognitive mechanisms.

## 1 Introduction

Everyday communication for young L2 learners rarely occurs in acoustically pristine environments. Classrooms bustle with peer chatter, hallways echo with background speech, and outdoor settings often contain environmental noise. Yet, primary school English instruction—particularly within the Chinese educational system—predominantly operates under quiet, controlled conditions, creating a stark mismatch between the learning context and the ecological reality of real-world listening. This pedagogical disconnect is especially consequential given that background noise disproportionately impairs speech perception for non-native compared to native listeners, a phenomenon well-documented at the sentence, word, and phonemic levels (Alqattan and Turner, 2021; Peng and Wang, 2016; Scharenborg and van Os, 2019; Shimizu et al., 2002; Zinszer et al., 2019). For instance, while native English listeners maintain relatively high speech recognition accuracy even at −5 dB signal-to-noise ratio (SNR), native Chinese listeners exhibit a steep decline when identifying English speech under comparable noise conditions (Simantiraki et al., 2023). Consequently, children who demonstrate adequate phonetic comprehension in quiet classrooms may nevertheless struggle to decode L2 speech in authentic, noisy communicative settings—a vulnerability that current curricula largely fail to address.

The theoretical mechanisms underlying this non-native disadvantage in noise remain a subject of active debate. On one hand, the experience-dependent hypothesis attributes the deficit to insufficient exposure to L2 speech in acoustically challenging environments. Cross-sectional evidence indicates that Dutch speakers with frequent daily English use outperform Chinese and Spanish speakers in perceiving English speech in noise (Cooke et al., 2008; Escudero et al., 2009; Tremblay et al., 2021), and longitudinal studies of immigrants demonstrate that length of residence in an English-speaking country correlates with improved L2 speech perception across both quiet and noisy conditions (Flege and Liu, 2001; Sagdiç, 2021). Mi et al. (2016) further reported that although a short-term study-abroad experience did not enhance Chinese university students' English vowel identification in quiet, it significantly improved their performance in noise, underscoring the critical role of complex acoustic experience (Cooke and García Lecumberri, 2018, 2019; Leong et al., 2018). Laboratory training studies provide converging evidence that incorporating multi-talker babble during perceptual training can improve noise-involved perception and promote generalization across acoustic conditions (Guediche et al., 2014; Mi et al., 2021). On the other hand, the L1 transfer hypothesis posits that native-language phonological categories fundamentally shape L2 perceptual strategies (Smit and Rathcke, 2025; Heinrich et al., 2010; Fuhrmeister et al., 2023), suggesting that the acoustic cues weighted by L1 experience may continue to influence—or interfere with—L2 processing even under adverse listening conditions. These two hypotheses generate competing predictions: whereas the former implies that noise merely amplifies an experience deficit that can be remedied through exposure, the latter raises the possibility that noise fundamentally alters the architecture of cross-linguistic transfer, forcing learners to abandon L1-tuned strategies. Which theoretical account holds for child learners remains empirically unresolved.

Compounding this theoretical ambiguity is a critical demographic gap: the vast majority of existing studies have involved adult learners, leaving the developmental trajectory of L2 speech-in-noise perception during childhood largely unexplored. The primary school years represent a period during which children exhibit greater neural and behavioral plasticity in response to language experience than adults (Werker and Tees, 1984; Kuhl, 2004; Werker and Hensch, 2015). Compared to adults, Japanese (Oh et al., 2011; Shafer et al., 2021) and Korean (Chung et al., 2010) children show more substantial gains in English vowel discrimination after equivalent lengths of immersion, suggesting that childhood may offer unique windows for L2 phonetic acquisition. Crucially, however, children's cognitive architecture differs qualitatively from that of adults in ways that directly impinge upon speech-in-noise processing. Auditory selective attention, working memory capacity, and executive control over phonological representations are not yet fully mature in 10- to 13-year-olds (Ginzburg et al., 2022; Porto et al., 2023), meaning that children may be less able than adults to deploy compensatory strategies to counteract noise interference. Consequently, the adult-derived conclusion that “noise impairs non-native speech perception” may manifest via fundamentally different mechanisms in children, and pedagogical recommendations based on adult data may be developmentally inappropriate.

A second, and equally pressing, theoretical lacuna concerns the boundary conditions of L1 influence on L2 speech perception under noise. Native language experience shapes individuals' speech categorization abilities (Smit and Rathcke, 2025) and modulates their preference for acoustic cues—for example, native Mandarin speakers rely more heavily on duration cues when identifying English vowels while underutilizing critical formant information (Heinrich et al., 2010; Fuhrmeister et al., 2023). Under quiet conditions, such L1-tuned perceptual strategies may facilitate or interfere with L2 identification depending on cross-linguistic acoustic similarity. Yet, two competing hypotheses can be advanced regarding noisy contexts. The persistent transfer hypothesis predicts that L1 phonological skills constitute domain-general auditory expertise that should transfer to L2 perception regardless of acoustic background, implying that children with stronger L1 vowel identification should consistently outperform their peers in L2 tasks. Conversely, the perceptual independence hypothesis proposes that noise disrupts the acoustic cues upon which L1 categories depend, forcing listeners to resort to L2-specific noise-adaptation strategies (e.g., selective attention to core formants) that are not scaffolded by L1 experience (Mattys et al., 2012; Drozdova et al., 2021). To date, no study has directly tested these competing predictions within a single pediatric sample by comparing the L1 → L2 predictive relationship across quiet and noisy conditions, leaving the theoretical boundary of L1 transfer empirically undefined.

Methodologically, prior investigations have relied almost exclusively on traditional parametric techniques—such as analysis of variance (ANOVA) and linear regression—to model the effects of acoustic background, developmental stage, and L1 ability on L2 perception. While these approaches are effective for detecting main effects and simple interactions, they impose restrictive assumptions of linearity and additive independence among predictors (Rimpler et al., 2025). Speech perception in noise, however, is governed by potential non-linear associations and interactions among acoustic condition, grade, and L1 identification performance: the impact of noise intensity on perceptual accuracy may increase non-linearly, while the facilitative effect of learning experience may saturate or attenuate as task difficulty escalates. Traditional linear models are ill-equipped to capture such dynamics or to quantify the marginal contributions of interacting features. Recent advances in interpretable machine learning—particularly ensemble methods such as random forests combined with SHapley Additive exPlanations (SHAP) values—offer a powerful alternative, enabling the modeling of non-linear relationships and the visualization of feature interactions without sacrificing interpretability (Breiman, 2001; Balan et al., 2023). Integrating these tools with behavioral experimentation thus provides a methodologically robust pathway to disentangle the intertwined influences of noise, development, and native-language proficiency, complementing conventional linear models by allowing flexible estimation of non-linear relationships and interactions.

To address these theoretical and methodological gaps, the present study focused on Chinese fifth- and sixth-grade children and their perception of English back vowels (/ɑ/, /ɔ/, /o/, /ʌ/, /u/, /℧/)—a phonetic category that is particularly challenging for native Mandarin speakers owing to substantial cross-linguistic acoustic discrepancies (Lai, 2010; Li and Woore, 2021). Back vowels are especially vulnerable to energetic masking in multi-talker noise because their distinguishing formant cues (particularly F1 and F2) often fall within the spectral regions most heavily masked by speech babble. Participants completed an English back-vowel identification task under quiet, 5 dB SNR, and −5 dB SNR multi-talker babble conditions, alongside a Chinese vowel identification task in quiet. By operationalizing school-based English learning experience through grade-level differences (fifth vs. sixth grade), and by systematically comparing L1 predictive effects across acoustic backgrounds, this design allows for a nuanced examination of how developmental experience and native-language proficiency interact with ecological listening demands.

Specifically, this study aimed to address three core questions: Does school-based English learning experience exert a bounded effect on English vowel identification across different acoustic backgrounds, and does the magnitude of the grade effect diminish as noise intensity increases? Does the predictive relationship between L1 vowel identification and L2 vowel identification differ between quiet and noisy conditions, thereby adjudicating between the persistent transfer and perceptual independence hypotheses? How can the interactive effects of acoustic background, grade, and L1 perception on L2 vowel identification be quantitatively characterized, and do non-linear machine learning models reveal patterns invisible to traditional linear approaches?

This investigation offers three specific contributions. Theoretically, it explores the acoustic boundary conditions of native-language influence on L2 vowel identification by examining whether L1 vowel-identification performance measured in quiet predicts L2 performance across quiet and noisy listening contexts, providing indirect empirical clues for evaluating competing hypotheses regarding L1-to-L2 transfer under adverse acoustic conditions. Methodologically, it introduces interpretable machine learning (random forests with SHAP analysis) to the study of pediatric L2 speech-in-noise perception, complementing conventional linear models by allowing flexible estimation of non-linear relationships and interactions and enabling precise quantification of non-linear feature interactions. Practically, the resulting feature-importance hierarchies and interaction profiles suggest candidate instructional approaches that warrant further empirical testing.

## 2 Methodology

### 2.1 Participants

Convenience sampling was used to select 30 children from each of Grades 5 and 6 (10–13 years old) from two primary schools representing different educational contexts in Taiyuan, Shanxi Province (one key urban primary school and one ordinary suburban primary school). The inclusion criteria were as follows: no participation in extracurricular English training, no use of English learning apps, and no experience of living in English-speaking countries; No professional music training; Normal hearing function with binaural hearing thresholds below 20 dB in the frequency range of 250–8,000 Hz; Daily family communication language was pure Mandarin or Mandarin mixed with Taiyuan dialect (pure dialect environments were excluded).

A total of 52 valid participants completed all experiments, including 25 in Grade 5 (12 boys, 13 girls, aged 10.2 ± 0.6 years old; 23 in pure Mandarin environment, two in Mandarin + Taiyuan dialect environment) and 27 in Grade 6 (13 boys, 14 girls, aged 11.8 ± 0.5 years old; 24 in pure Mandarin environment, three in Mandarin + Taiyuan dialect environment). The baseline information of the participants is shown in Table 1. There was no significant difference in the English final exam scores of the two groups of children last semester (*p* > 0.05), but there were significant grade differences in age, English learning duration, and weekly class hours (*p* < 0.001). All participants volunteered to participate and received small stationery gifts after the experiment.

**Table 1:** Baseline participant characteristics (M ± SD).

Characteristic	Grade 5 (*n* = 25)	Grade 6 (*n* = 27)	*t*	*p*
Age (years)	10.2 ± 0.6	11.8 ± 0.5	−10.48	<0.001
Duration of English learning (years)	2.1 ± 0.3	3.2 ± 0.4	−11.24	<0.001
Weekly English class hours (sessions)	3.0 ± 0.0	4.0 ± 0.0	–	<0.001
Last semester's English final exam score (score)	85.6 ± 6.2	88.3 ± 5.8	−1.87	>0.05

Weekly English class hours were fixed at three sessions for all fifth-grade participants and four sessions for all sixth-grade participants (no within-group variability). The group difference was confirmed by an exact test; no standard *t*-statistic is reported because the pooled variance is undefined when one or both groups have zero variance. The *p*-value reflects a two-sample exact test for location shift.

### 2.2 Experimental materials

#### 2.2.1 Vowel stimulation

For Chinese vowels, 20 stimuli (/ɑ1-4/, /ɤ1-4/, /i1-4/, /u1-4/, /y1-4/) were generated by combining four tones with five Mandarin monophthongs (/ɑ/, /ɤ/, /i/, /u/, /y/), comprehensively covering the core tonal variants of Chinese vowels (Lai, 2010). The selection of English vowels was based on the identification difficulties of native Chinese speakers, which are particularly pronounced in child populations—studies have shown that fifth-grade Chinese children achieve an identification accuracy of only 45%−50% for /ʌ/ and /℧/ (Li and Woore, 2021). Accordingly, six English back vowels (/ɑ/, /ɔ/, /o/, /ʌ/, /u/, /℧/) with significant acoustic differences from the Chinese vowel system were selected (Lai, 2010; Li and Woore, 2021); Table 2 summarizes acoustic properties of the English experimental stimuli. Both types of vowels were embedded in /hVd/ syllables and recorded by native speakers of the respective languages: Chinese vowels were pronounced by native speakers of standard Mandarin, and English vowels by native speakers of American English, with a sampling rate of 44.1 kHz and 16-bit quantization. Praat software was used to remove the consonant segments at the beginning and end of each syllable, retaining a 170-ms pure vowel segment in the middle. The fundamental frequency onset of all vowels was standardized to ensure the acoustic consistency of the stimulus materials.

**Table 2:** Acoustic properties of target English back vowels.

English vowel	Reference Mandarin category	F1 (Hz)	F2 (Hz)	Duration (ms)	Acoustic description
/ɑ/	/ɑ/	730	1,090	170	Lower F2, relatively retracted tongue position
/ɔ/	/o/	570	840	170	Higher F1, relatively large mouth opening
/o/	/o/	440	1,020	170	Higher F2, relatively advanced tongue position
/ʌ/	/ɤ/	660	1,190	170	Distinct tongue position from reference category
/u/	/u/	300	870	170	Lower F2, rounded lip configuration
/℧ /	/u/	380	950	170	Relatively large mouth opening

Values represent measurements for English stimuli only. Reference Mandarin Category is provided for phonological reference and does not represent within-study acoustic comparison with Mandarin tokens. Formant values were extracted from the central 170-ms steady-state portion of each /hVd/ token using Praat.

#### 2.2.2 Background noise

Twelve-talker speech noise was adopted as the background noise, with the preparation procedure as follows: audio recordings were made of six native English speakers (three males and three females) reading the children's book *The Cat in the Hat*, with each recording lasting 5 min; each audio clip was subjected to loudness normalization [with a root mean square (RMS) value of 0.01] and then mixed to generate six-talker speech noise; Two segments of six-talker speech noise were randomly selected for secondary mixing to obtain twelve-talker speech noise; 400-ms segments were randomly extracted from the twelve-talker speech noise to ensure consistent spectral distribution across all segments; English vowel stimuli were embedded in the center of the noise segments (with vowels starting at 115 ms and ending at 285 ms of the noise duration), and experimental stimuli with two noise intensities (5 dB SNR and −5 dB SNR) were generated separately.

### 2.3 Experimental procedure

#### 2.3.1 Pre-experimental preparation

A 20-min standardized phonetic training session was administered, with the procedures as follows: Chinese vowel Pinyin forms (e.g., ā, á) and their corresponding English vowel /hVd/ words (e.g., Hod, Hawed) were presented sequentially, with each stimulus displayed for 3 s; The experimenter read each stimulus aloud three times (with tone demonstrations for Chinese vowels and phonetic symbol explanations for English vowels); Participants repeated each stimulus twice, and the experimenter corrected pronunciation deviations in real time; Upon completion of the training, a proficiency test with 10 trials was conducted. Participants who failed to meet the proficiency criterion (test accuracy <100%) received an additional 10-min targeted phonetic training and were required to pass a retest before proceeding to the formal experiment. A total of three participants met the proficiency criterion after the secondary training. The remaining five children from the initial pool of 60 were excluded due to failure to meet the proficiency criterion or voluntary withdrawal, yielding the final sample of 52 participants.

#### 2.3.2 Orthogonal experiment

The experiment was controlled using Sykofizx software (Tucker-Davis Technologies (TDT), Alachua, FL, USA). Speech stimuli were processed with a portable digital filter (RM1) and presented to participants' right ears via AKG-K240 over-ear headphones, with the target stimulus intensity calibrated to 70 dB (calibrated using an AEC201-AIEC60318 artificial ear and a Larson-Davis Model2800 sound level meter). Stimuli were presented monaurally to the right ear. This presentation mode was adopted to eliminate inter-participant variability arising from minor inter-aural hearing differences and keep auditory input pathways consistent across all participants. We note that monaural listening differs from natural binaural classroom listening, which imposes certain limits on the ecological generalization of behavioral outcomes. The experiment consisted of two tasks:

Chinese Vowel Identification Task: this was a closed-set identification task. The task included 20 response options corresponding to the 20 Mandarin vowel-tone combinations, visually presented on-screen as Pinyin forms. Participants selected one target option via mouse click for each trial. Response-option positions were randomized across individual trials to eliminate positional response bias. A 10-s response deadline was enforced for every trial; participants were required to make a selection within this time window. There were 10 trials for each vowel-tone combination, resulting in a total of 200 trials presented in random order with an inter-stimulus interval of 2 s.

English Vowel Identification Task: this was also a closed-set identification task, with six on-screen displayed response options corresponding to the six English /hVd/ target words. Participants made their choice by mouse click. Response-option positions were randomized across trials. Consistent with the Mandarin task, a 10-s response deadline was applied for each trial. There were 15 trials set for each vowel across three acoustic backgrounds (quiet, 5 dB SNR, −5 dB SNR), yielding a total of 270 trials (90 trials per background). A 5-min practice session was conducted prior to the formal experiment, which included 10 trials in quiet conditions and 10 trials in 5 dB SNR noise, with real-time feedback provided to familiarize participants with the various acoustic backgrounds. A 1-min break was scheduled every 10 min throughout the experiment, with the total experimental duration being approximately 60 min. The software automatically recorded participants' response selections and reaction times. All English trials, pooling across vowels and acoustic backgrounds, were fully randomized for each participant.

### 2.4 Data processing

#### 2.4.1 Basic data processing

Raw data were exported from Sykofizx software, and trials with reaction times of less than 200 ms or more than 10,000 ms (accounting for less than 1% of the total data) were excluded using Excel. The vowel identification accuracy under each condition (i.e., the mean accuracy of all trials for a single vowel) was then calculated. Subsequent statistical analyses were performed using SPSS 22.0 (IBM Corp., Armonk, NY, USA), with the following procedures: a two-way repeated-measures analysis of variance (ANOVA) was conducted with dialect type as the covariate to examine the main effects and interaction effect of grade (between-subjects factor) and acoustic background (within-subjects factor); Pearson correlation analysis was applied to explore the correlation between Chinese and English vowel identification; Multiple regression analysis was used to verify the predictive effect of native language perception on second-language perception, with the significance level set at α = 0.05.

#### 2.4.2 Traditional machine learning modeling

##### 2.4.2.1 Data preprocessing

Categorical variables, including grade, gender, acoustic background, and dialect type, were numerically encoded. Continuous variables, including English-learning duration, weekly English class hours, Chinese and English vowel-identification accuracy, and reaction time, were scaled to the [0, 1] interval using Min-Max normalization. To prevent information leakage, the data were first partitioned at the level of the original participants. The 52 participants were assigned to a training set (*n* = 36), a validation set (*n* = 11), and a test set (*n* = 5) using a grade-stratified split with a fixed random seed (seed = 42). All three acoustic-background observations from a given participant were retained in the same partition. The normalization parameters were estimated exclusively from the training set and subsequently applied, without refitting, to the validation and test sets. No bootstrap resampling, synthetic noise injection, or other data augmentation was performed. The models were therefore trained, tuned, and evaluated exclusively using the original observations. The unit of analysis was the participant-by-acoustic-background observation, yielding 156 observations in total (52 participants × 3 acoustic backgrounds), while participant-level grouping was preserved throughout data splitting and model evaluation.

##### 2.4.2.2 Model construction

Linear Regression Model: constructed using the least squares method, with English vowel identification accuracy as the dependent variable and acoustic background, grade, dialect type and other factors as independent variables. The standardized coefficients (β) were used to accurately quantify the linear contribution of each variable, and no decision tree-related parameters were required. Random Forest Model: the parameter settings were as follows: 100 decision trees, maximum tree depth of 5, minimum sample split size of 10, and Gini coefficient for feature selection. The ensemble strategy of “average output” was adopted, and SHAP values were integrated to quantify the marginal contributions of features. The significance of mean SHAP values was assessed via bootstrap confidence intervals (1,000 resamples); values whose 95% CI excluded zero were deemed significant (Balan et al., 2023).

##### 2.4.2.3 Model evaluation

A comprehensive comparative analysis of model performance was conducted using three metrics: the coefficient of determination (*R*^2^), root mean square error (RMSE), and mean absolute error (MAE).

## 3 Results

### 3.1 Effects of school-based English learning on English vowel identification in Chinese children under different acoustic background conditions

Descriptive statistics including group means, standard deviations, and inferential test statistics for each grade and acoustic-background combination are reported in Table 3. Descriptive results showed that the English vowel identification performance of children in all grades decreased gradually with the increase in acoustic task difficulty, and older children exhibited overall superior performance across all acoustic conditions compared to younger children. Reaction times were prolonged with increasing noise intensity, with sixth-grade children demonstrating shorter mean reaction times than fifth-grade children. Furthermore, the grade differences between the two groups of children narrowed gradually with the increase in acoustic task difficulty.

**Table 3:** English vowel identification accuracy (M ± SD, %) and reaction time (ms) by grade and acoustic background.

Acoustic background	Grade 5 accuracy (%)	Grade 6 accuracy (%)	Accuracy mean difference (%)	Accuracy statistics	Grade 5 RT (ms)	Grade 6 RT (ms)	RT mean difference (ms)	RT statistics
Quiet	62.3 ± 8.5	75.6 ± 7.2	13.30	*t* = 6.06, *p* < 0.01	1,250 ± 150	1,080 ± 120	170	*t* = 4.49, *p* < 0.01
5 dB SNR noise	54.1 ± 9.2	63.9 ± 8.6	9.80	*t* = 3.96, *p* < 0.01	1,480 ± 180	1,320 ± 160	160	*t* = 3.38, *p* < 0.01
−5 dB SNR noise	48.7 ± 10.3	51.9 ± 9.8	3.20	*t* = 1.15, *p* > 0.05	1,620 ± 200	1,510 ± 190	110	*t* = 2.03, *p* < 0.05

All pairwise comparisons were treated as planned contrasts. The Bonferroni–Holm sequential correction was applied to control family-wise Type-I error across the six pairwise tests. Reported *p*-values are corrected *p*-values.

A two-way repeated-measures analysis of variance (ANOVA) was conducted on English vowel identification accuracy, with grade (Fifth Grade, Sixth Grade) as the between-subjects variable, acoustic background (Quiet, 5 dB SNR, −5 dB SNR) as the within-subjects variable, and dialect type as the covariate. The results showed that Mauchly's Test of Sphericity was non-significant (Mauchly's *W* = 0.92, *p* > 0.05), so no correction was required. The main effect of the covariate (dialect type) was non-significant [*F*_(1, 50)_ = 0.87, *p* > 0.05]. There was a significant main effect of acoustic background [*F*_(2, 100)_ = 42.36, *p* < 0.001, partial η^2^ = 0.46], a significant main effect of grade [*F*_(1, 50)_ = 18.72, *p* < 0.001, partial η^2^ = 0.27], and a significant interaction effect between acoustic background and grade [*F*_(2, 100)_ = 4.18, *p* < 0.05, partial η^2^ = 0.08]. Results of simple effect analysis with Bonferroni correction for multiple comparisons indicated that sixth-grade children's English vowel identification performance was significantly higher than that of fifth-grade children under the quiet condition (*p* < 0.001), 5 dB SNR noise condition (*p* < 0.01), and −5 dB SNR noise condition (*p* < 0.05). A one-way ANOVA (with dialect type as the covariate) was performed to examine the grade effect on Chinese vowel identification. The results revealed that the main effect of grade was non-significant [*F*_(1, 50)_ = 0.41, *p* = 0.526, partial η^2^ = 0.01], and the main effect of the covariate (dialect type) was also non-significant (*F* = 0.62, *p* > 0.05). Fifth-grade children achieved a Chinese vowel identification accuracy of 88.3 ± 5.6%, while sixth-grade children had an accuracy of 89.1 ± 4.9%.

Across acoustic conditions, identification accuracy declined and reaction time became longer as noise intensity increased. Sixth-grade children exhibited higher identification accuracy and shorter reaction times relative to fifth-grade children in all listening contexts. The magnitude of grade-related advantages for both accuracy and reaction time diminished as background-noise intensity rose: the accuracy gap between grades was largest under quiet conditions and became markedly smaller at −5 dB SNR; similarly, reaction-time differences between grades narrowed with increasing noise.

### 3.2 Effects of native language phonetic perception on English phonetic perception under different acoustic background conditions

Results of the Pearson correlation analysis are presented in Table 4. Under the quiet condition, Chinese vowel identification was significantly correlated with English vowel identification in the same condition (*r* = 0.37, *p* < 0.01); this correlation was more pronounced for vowels with a high degree of formant overlap (e.g., /ɑ/, /u/) (*r* = 0.42, *p* < 0.01). Chinese vowel identification showed no significant correlation with English vowel identification in the 5 dB SNR condition (*r* = 0.21, *p* > 0.05) or the −5 dB SNR condition (*r* = 0.03, *p* > 0.05). Furthermore, a significant positive correlation was found in English vowel identification between the two SNR noise conditions (*r* = 0.46, *p* < 0.01); significant correlations were also observed between English vowel identification under the quiet condition and that under the 5 dB SNR noise condition (*r* = 0.45, *p* < 0.01), as well as between the quiet condition and the −5 dB SNR noise condition (*r* = 0.44, *p* < 0.01).

**Table 4:** Correlation matrix for vowel identification tasks.

Measure	1. Quiet (Chinese) vowel identification	2. Quiet (English) vowel identification	3. 5 dB SNR noise English identification	4. −5 dB SNR noise English identification
1. Quiet (Chinese) vowel identification	1.00	0.37^**^	0.21	0.03
2. Quiet (English) vowel identification		1.00	0.45^**^	0.44^**^
3.5 dB SNR Noise English Identification			1.00	0.46^**^
4. −5 dB SNR Noise English identification				1.00

^**^p < 0.01.

Results of the multiple regression analysis are presented in Table 5. In Step 1, gender, grade, and dialect type were entered as control variables into the regression equation. The results showed that grade had a significant predictive effect on English vowel identification under all three acoustic background conditions (*p*s <0.05), whereas gender and dialect type exerted no significant predictive effects (*p*s > 0.05). In Step 2, Chinese vowel identification under the quiet condition was added to the regression equation. The results indicated that after controlling for the aforementioned variables, Chinese vowel identification under the quiet condition significantly and positively predicted English vowel identification under the quiet condition (*p* < 0.05), but had no predictive effect on English vowel identification under the two noise conditions (*p*s > 0.05).

**Table 5:** Hierarchical regression predicting English vowel identification.

Model	Step	Predictors	*b*	SE	β	*t*	*p*	Adjusted *R*^2^
Model 1 (Quiet)	Step 1	Grade	13.82	4.50	0.47	3.07	<0.01	0.22
		Gender	−6.23	5.05	−0.21	−1.23	>0.05	
		Dialect type	1.52	2.10	0.08	0.72	>0.05	
	Step 2	Grade	12.78	4.65	0.44	2.75	<0.01	0.33
		Gender	−7.93	4.73	−0.28	−1.68	>0.05	
		Dialect type	1.36	2.05	0.07	0.66	>0.05	
	Chinese vowel identification		0.46	0.20	0.46	2.30	<0.05	
Model 2 (5 dB)	Step 1	Grade	10.12	3.48	0.49	2.91	<0.01	0.23
		Gender	−3.06	3.52	−0.15	−0.87	>0.05	
		Dialect type	0.98	1.85	0.06	0.53	>0.05	
	Step 2	Grade	9.76	4.49	0.48	2.17	<0.05	0.24
		Gender	−3.65	3.55	−0.18	−1.03	>0.05	
		Dialect type	0.82	1.80	0.05	0.45	>0.05	
	Chinese vowel identification		0.16	0.15	0.18	1.07	>0.05	
Model 3 (−5 dB)	Step 1	Grade	3.36	1.29	0.44	2.60	<0.05	0.24
		Gender	−2.36	1.30	−0.31	−1.82	>0.05	
		Dialect type	0.56	1.25	0.04	0.45	>0.05	
	Step 2	Grade	3.33	1.32	0.44	2.52	<0.05	0.24
		Gender	−2.33	1.31	−0.30	−1.78	>0.05	
		Dialect type	0.04	0.43	0.02	0.20	>0.05	
	Chinese vowel identification		0.04	0.20	0.04	0.20	>0.05	

### 3.3 Traditional machine learning predictive models for English vowel identification in Chinese children under varying acoustic conditions

To precisely examine the associations among various factors on English vowel identification, this study constructed two traditional machine learning models—linear regression and random forest—and compared their performance on the original 156 participant-by-background observations (52 participants × 3 acoustic backgrounds), using strict participant-level train/validation/test splitting without any data augmentation. The results (see Table 6) demonstrated that the random forest model outperformed the linear regression model in predictive accuracy by a significant margin.

**Table 6:** Performance comparison of linear regression and random forest models across different acoustic backgrounds.

Model	Acoustic background	*R* ^2^	RMSE	MAE
Linear regression	Quiet	0.33	0.085	0.068
	5 dB SNR noise	0.24	0.092	0.075
	−5 dB SNR noise	0.24	0.095	0.078
	Average performance	0.27	0.091	0.074
Random forest	Quiet	0.56	0.058	0.045
	5 dB SNR noise	0.54	0.061	0.048
	−5 dB SNR noise	0.50	0.065	0.052
	Average performance	0.53	0.061	0.048

In terms of overall performance, the coefficient of determination (*R*^2^) of the random forest model reached 0.53, representing a 96.3% increase compared with 0.27 for the linear regression model. The root mean square error (RMSE) and mean absolute error (MAE) of the random forest model decreased to 0.061 and 0.048, respectively, corresponding to reductions of 33.0 and 35.1% relative to the linear regression model (RMSE = 0.091, MAE = 0.074). The random forest's better fit under noisy conditions likely reflects predictive interaction patterns in the behavioral data that the specified linear model did not capture in this sample. This pattern is consistent with the possibility that the impact of acoustic background on identification increases with noise intensity while the grade effect attenuates, though the cross-sectional design and modest sample size preclude inferences about underlying cognitive mechanisms. This interaction was not detected by the linear regression model in the present sample, suggesting that the additive, independent-effects structure of linear regression may be insufficient to represent the observed grade-by-noise association in this dataset. The results (Table 6) showed that the random forest model achieved higher predictive accuracy than the linear regression model across all acoustic backgrounds. The random forest *R*^2^ reached (0.50–0.56), compared with (0.24–0.33) for linear regression. Although the absolute advantage is more conservative than in the original analysis—reflecting the removal of augmentation and strict out-of-participant validation—the random forest continued to show a relatively better fit in this sample (Rimpler et al., 2025).

Linear regression quantified the linear contributions of features using standardized coefficients (Table 7). The results indicated that acoustic background (standardized coefficient = 0.29) was the primary linear factor associated with English vowel identification, followed by grade (0.29) and Chinese vowel identification accuracy (0.21). English learning duration (0.16) exhibited a significant effect, whereas weekly English class hours (0.12) and gender (0.06) did not reach statistical significance (all *p*s > 0.05). However, linear regression only captures the independent linear effects of individual variables and fails to model feature interactions (Strobl et al., 2009).

**Table 7:** Standardized coefficients and significance of features for the linear regression model.

Predictors	Standardized coefficient	*t*	*p*
Acoustic background	0.29	3.82	<0.001
Grade	0.25	3.21	<0.01
Chinese vowel identification accuracy	0.21	2.75	<0.05
English learning duration	0.16	2.13	<0.05
Weekly English class hours	0.12	1.68	>0.05
Gender	0.06	0.89	>0.05
Dialect type	0.05	0.62	>0.05

The random forest model calculated feature importance based on the decrease in node impurity and quantified the marginal contributions of features using SHAP values (see Table 8). The results showed that the feature importance ranking was as follows: Acoustic Background (0.31) > Grade (0.25) > Chinese Vowel Identification Accuracy (0.19) > English Learning Duration (0.15) > Weekly English Class Hours (0.10) > Dialect Type (0.06) > Gender (0.05). This ranking was consistent with the order of linear contributions identified by the linear regression model; however, the random forest model further captured predictive interaction patterns not represented in the linear specification.

**Table 8:** Feature importance and SHAP values for the random forest model.

Predictors	Feature importance	Mean SHAP
Acoustic background	0.31	0.28
Grade	0.25	0.18
Chinese vowel identification accuracy	0.19	0.15
English learning duration	0.15	0.12
Weekly English class hours	0.10	0.09
Dialect type	0.06	0.05

Visualization of non-linear relationships: SHAP value analysis revealed that the contribution of acoustic background to prediction outcomes exhibited a non-linear increase with rising noise intensity (SHAP value = 0.20 in quiet conditions, 0.28 at an SNR of 5 dB, and 0.35 at an SNR of −5 dB). In contrast, the contribution of grade showed a non-linear decrease as noise intensity increased (SHAP value = 0.28 in quiet conditions, 0.17 at an SNR of 5 dB, and 0.09 at an SNR of −5 dB). Additionally, the marginal contribution of L1 vowel identification accuracy (Chinese vowel identification) decreased across increasingly adverse listening conditions: the average SHAP value was 0.23 in quiet conditions (*p* < 0.05), 0.10 at 5 dB SNR (*p* > 0.05), and only 0.03 at −5 dB SNR (*p* > 0.05). This indicates a significant non-linear interaction between L1 perception and acoustic background—L1 perception exerts a significant predictive effect only in quiet environments, and its contribution is almost negligible in high-noise environments. These findings visually validate the core patterns that “the grade effect weakens as background noise intensity increases” (Li and Woore, 2021; Reh et al., 2021) and “the L1 transfer effect is constrained by acoustic background” (Mattys et al., 2009; Calcus, 2024).

Both traditional machine learning models validated the predictive boundary of native-language perception (see Table 9): Chinese vowel identification accuracy exerted a significant positive predictive effect on English vowel identification only under quiet conditions, with no predictive power observed in noisy backgrounds.

**Table 9:** Predictive effects of Chinese vowel identification accuracy across two models and different acoustic backgrounds.

Model	Acoustic background	Feature importance/SHAP value	*t/Z*	*p*
Linear regression	Quiet	0.46	2.30	<0.05
	5 dB SNR noise	0.18	1.07	>0.05
	−5 dB SNR noise	0.04	0.20	>0.05
Random forest	Quiet	0.23	2.45	<0.05
	5 dB SNR noise	0.10	1.12	>0.05
	−5 dB SNR noise	0.03	0.31	>0.05

Specifically, in the linear regression model, the standardized coefficient for Chinese vowel identification accuracy was 0.46 under quiet conditions (*p* < 0.05), whereas it was non-significant at an SNR of 5 dB (0.18, ^*^p^*^ > 0.05) and an SNR of −5 dB (0.04, *p* > 0.05). In the random forest model, the average SHAP value of Chinese vowel identification accuracy reached 0.23 under quiet conditions (*p* < 0.05), while its contributions were weak and non-significant at an SNR of 5 dB (0.10, ^*^p^*^ > 0.05) and an SNR of −5 dB (0.03, *p* > 0.05). These results further suggest that the mechanism underlying English speech perception in noisy environments is associated with processes independent of native-language perception (Wang and Xu, 2021).

## 4 Discussion

### 4.1 The impact of school English learning experience on English vowel identification across different acoustic backgrounds

This study found that sixth-grade children significantly outperformed fifth-grade children in English vowel identification accuracy under both quiet and noisy conditions, and that the grade effect gradually diminished as acoustic task difficulty increased. These results indicate a cross-sectional association between grade-related learning experience and English-vowel identification performance among the participating children.

However, the interaction between grade and acoustic background reveals a critical boundary: the effectiveness of regular school English instruction is attenuated in complex acoustic environments. Under quiet conditions, the accuracy difference between grades reached 13.30%, whereas at −5 dB SNR it narrowed to merely 3.20%. This observed convergence does not uniquely support an attentional-resource explanation. One possible account draws on developmental cognitive load theory: unlike adult L2 learners, who can recruit mature auditory selective attention and phonological working memory to compensate for noise interference (Mattys et al., 2009), primary-school children's executive control over auditory attention is not yet fully developed (Ginzburg et al., 2022; Porto et al., 2023). As background noise intensifies, both age groups must allocate disproportionate cognitive resources to suppress masking, leaving fewer attentional resources available to leverage their linguistic experience advantages. An alternative interpretation is that increasing noise imposes a shared developmental bottleneck in auditory segregation and the processing of degraded spectral information for both grade groups, thereby reducing the relative benefit associated with greater classroom experience (Calcus, 2024).

Furthermore, the absence of a significant grade effect in Chinese vowel identification (fifth grade: 88.3%; sixth grade: 89.1%) indicates that the experiential advantage is specific to the L2 system. Native-language speech perception has already stabilized by the upper-primary years and is unaffected by grade progression or dialect background. This dissociation—grade effects observed exclusively at the L2 level—underscores the high plasticity and strong experience-dependence of the L2 speech system during childhood, while confirming that L1 phonological representations may be relatively stable.

### 4.2 The predictive role and boundaries of native-language speech perception in English vowel identification

The results indicate that Mandarin vowel-identification performance measured in quiet positively predicted English vowel identification under quiet conditions; however, this predictive effect disappeared entirely under the tested noise conditions. This finding provides evidence relevant to distinguishing between two competing theoretical accounts of L1–L2 perceptual relationships.

Under quiet conditions, a robust link exists between L1 and L2 speech perception, primarily because the speech signal is acoustically intact, allowing children to rely on shared auditory processing pathways (Wang and Xu, 2021). The basic phonetic discrimination abilities cultivated through Chinese vowel identification—such as sensitivity to fundamental frequency and formant transitions—can be transferred to English vowel identification tasks. This pattern is consistent with the Native Language Transfer Theory of second language acquisition. Further analysis revealed that the magnitude of this transfer is modulated by cross-linguistic acoustic similarity (Lai, 2010; Li and Woore, 2021): for vowels with high formant overlap between Mandarin and English (e.g., /ɑ/, /u/), the correlation was stronger (*r* = 0.42, *p* < 0.01), whereas for vowels lacking Mandarin phonemic counterparts (e.g., /ʌ/, /℧/), the transfer effect was weaker (*r* = 0.25, *p* > 0.05). This acoustic-similarity gradient suggests that L1 facilitation operates through shared phonetic feature detectors rather than through domain-general auditory acuity.

We refrain from positing a functionally independent L2 processing module for noisy listening, given the absence of Mandarin-in-noise data in the present dataset. When multi-talker babble disrupts the spectral and temporal cues upon which L1 categories depend, children can no longer rely on Mandarin-tuned perceptual strategies. Instead, English vowel identification in noise requires specific adaptation mechanisms—such as selective attention to noise-resistant core formants and suppression of redundant masking information (Mattys et al., 2012; Drozdova et al., 2021)—that are not scaffolded by L1 experience. The significant correlations observed between English vowel identification across different noise levels (*r* = 0.44–0.46, *p* < 0.01), coupled with the absence of correlation between L1 and L2-in-noise measures, indicates that L2 speech perception in adverse acoustic environments may rely on processes less strongly associated with quiet L1 performance of the native-language system. This pattern may reflect processes that are less strongly associated with L1 performance measured in quiet; however, the present data cannot determine whether L1 and L2 vowel identification rely on shared or distinct processes under matched noisy conditions.

### 4.3 Validation and complementation of factors influencing speech perception by machine learning models

The linear regression and random forest models constructed in this study quantitatively verified the associations among acoustic background, grade, and native-language perception. Under the corrected participant-level validation, the random forest model continued to show better predictive accuracy than linear regression model (Table 6). This advantage suggests that the fitted random forest captured non-linear predictive structure or interactions not represented in the particular linear specification. It does not establish underlying cognitive dynamics, nor does it imply a general limitation of linear modeling because alternative linear specifications could include non-linear transformations and interaction terms. SHAP patterns (Table 8) were consistent with attenuation of the predictive association between quiet-measured L1 performance and L2 identification as noise increased.

The better fit of the random forest model likely reflects non-linear predictive patterns in the behavioral data that the linear regression model did not capture in this sample. It should be emphasized that these are predictive associations rather than established cognitive mechanisms: the cross-sectional design and modest sample size (*N* = 52 independent participants) preclude strong inferences about underlying cognitive dynamics. The observed non-linearities may arise from interactions among acoustic background, grade, and L1 performance, but they require replication in larger, more diverse samples before they can be treated as stable phenomena. Specifically, linear regression identified acoustic background (β = 0.29), grade (β = 0.25), and Chinese vowel identification (β = 0.21) as significant linear predictors, but it did not detect interactions among these factors in this sample. The random forest model, coupled with SHAP analysis, revealed predictive interaction patterns: the estimated contribution of acoustic background increased non-linearly with noise intensity (SHAP = 0.20 in quiet, 0.28 at 5 dB, and 0.35 at −5 dB), while the contribution of grade decreased (SHAP = 0.28 in quiet, 0.17 at 5 dB, and 0.09 at −5 dB). The marginal contribution of L1 vowel identification also showed a graded decline across conditions (SHAP = 0.23 in quiet, 0.10 at 5 dB, and 0.03 at −5 dB). These SHAP profiles are consistent with the theoretical prediction that L1 transfer is constrained by acoustic background, but they should be treated as exploratory descriptive patterns rather than as confirmation of cognitive mechanisms, given the modest sample size and cross-sectional design (Zinszer et al., 2022; Bsharat-Maalouf and Karawani, 2022).

Based on the feature-importance ranking generated by the random forest model (Strobl et al., 2009), the present findings suggest several candidate instructional approaches that warrant evaluation in future intervention studies. Because the present study did not test any instructional intervention, these proposals should be regarded as research hypotheses rather than demonstrated teaching recommendations.

(1) Tiered noise-adaptation training. Future intervention studies could evaluate whether noise-adaptation training tailored to grade-related differences improves English vowel identification. For younger learners, studies might examine training that begins with moderate noise levels and uses vowels with relatively high cross-linguistic acoustic similarity, such as /ɑ/ and /u/. For older learners, studies could investigate progressively more difficult listening conditions and vowels without close Mandarin counterparts, such as /ʌ/ and /℧/. The effectiveness, optimal duration, frequency, and curricular integration of such training remain to be determined empirically.
(2) Formant-visualization-based instruction. Future studies could evaluate whether visualizing English-vowel formants using speech-analysis software such as Praat helps learners associate acoustic cues with vowel categories. For example, an intervention could compare the F1 and F2 patterns of English /ʌ/ and Mandarin /ɤ/ and examine whether this visualization facilitates vowel identification under quiet or noisy conditions. Controlled studies are needed to determine whether formant visualization improves learners' use of relevant acoustic cues.
(3) Differentiated experience-supplementation activities. Future research could test whether supplementary vowel-category activities or noise-cue discrimination tasks benefit learners with different amounts of English-learning experience. Candidate activities might include vowel-matching tasks, acoustic-cue matching, and vowel identification under different noise levels. However, the appropriate content, intensity, duration, and effectiveness of these activities should be established through controlled intervention studies.

## 5 Limitations

Although this study identifies several factors associated with English vowel-identification performance, it has three main limitations that should be addressed in future research.

First, convenience sampling was used, with only 52 independent participants recruited from only two primary schools in Taiyuan, predominantly children exposed to Mandarin or Mandarin + Taiyuan dialect. The limited coverage of geographical regions, school types, and dialect backgrounds means future studies should include children from a wider range of regions, school types, and dialect backgrounds to assess the generalizability of the findings. Because dialect backgrounds may alter L1 phonological categories and, consequently, the boundary of L1-to-L2 transfer, future studies should recruit geographically and linguistically diverse samples to cross-validate the perceptual independence hypothesis.

Second, the experimental materials were limited to English twelve-talker babble and English back vowels. The English speech masker used in this study was generated from read-aloud recordings of a single children's text rather than spontaneous conversational speech. While read-speech-based multi-talker babble affords high experimental control, it lacks lexical, prosodic, turn-taking and temporal variability characteristic of real-world classroom dialogue; accordingly, results may not directly generalize to natural classroom babble. Future work should adopt both controlled read-speech maskers and naturalistic or mixed-language conversational masker stimuli. Real-world Chinese primary-school classrooms often involve Chinese–English mixed noise (e.g., peer conversations in Mandarin alongside English instructional audio). The absence of mixed-language noise and other speech units (e.g., consonants, lexical tones, stress patterns) limits the ecological validity of the study. Future research should incorporate Chinese–English mixed babble and investigate whether the L1-independence observed here generalizes to other phonetic domains.

Third, Chinese vowel identification was only measured under quiet listening conditions in this study. We lack matched L1 identification data under noise, and therefore cannot rule out the possibility that Mandarin vowel performance under noise would correlate with English-in-noise identification. Future research should collect acoustically matched L1 and L2 vowel identification data within the same sample to directly test L1–L2 relationships under noisy listening conditions.

## 6 Future research

Complementary neurophysiological evidence could further illuminate processing mechanisms underlying vowel identification in noise. Event-related potentials (ERPs) could be used to examine whether the N1 (early auditory encoding) and P2 (phonetic categorization) components during English vowel identification in noise differ as a function of L1 vowel ability, clarifying the processing stages at which L1 performance may be associated with L2 vowel identification under adverse listening conditions at early sensory or later decisional processing stages (Rizzi and Bidelman, 2024). Eye-tracking could further illuminate how children allocate visual attention to articulatory cues when auditory information is degraded (Grieco-Calub et al., 2025). Such neurophysiological measurements would complement the present behavioral dataset.

## 7 Conclusion

Among 52 Mandarin-speaking fifth- and sixth-grade children, English vowel-identification accuracy decreased and reaction time increased as listening conditions became noisier. Sixth graders performed better overall, although the grade-associated accuracy advantage narrowed with increasing noise intensity. Mandarin vowel-identification accuracy measured in quiet predicted English performance in quiet but showed no significant predictive association with English vowel identification under the two noise conditions. Random-forest analyses suggested non-linear predictive patterns and possible feature interactions that complemented the specified linear model; these modeling patterns should not be interpreted as direct evidence of non-linear cognitive mechanisms or as universal limits on linear modeling. Despite participant-level data partitioning and the absence of bootstrap data augmentation, the modest number of independent participants (*n* = 52) limits the stability and generalizability of the model estimates. Larger independent samples are needed to replicate the predictive patterns and evaluate candidate instructional applications.

## Data Availability

The original contributions presented in the study are included in the article/supplementary material, further inquiries can be directed to the corresponding author.
